# Enhanced Defluoridation Capacity From Aqueous Media via Hydroxyapatite Decorated With Carbon Nanotube

**DOI:** 10.3389/fchem.2018.00104

**Published:** 2018-04-11

**Authors:** Qingzi Tang, Tongdan Duan, Peng Li, Ping Zhang, Daishe Wu

**Affiliations:** ^1^Key Laboratory of Poyang Lake Environment and Resource Utilization, School of Environmental and Chemical Engineering, Ministry of Education, Nanchang University, Nanchang, China; ^2^Australian Institute for Bioengineering and Nanotechnology, The University of Queensland, Brisbane, QLD, Australia

**Keywords:** fluoride removal, hydroxyapatite decorated with carbon nanotube (CNT-HAP), ion-exchanged, hydroxyl anions, removal mechanism

## Abstract

In this work, the potential of a novel hydroxyapatite decorated with carbon nanotube composite (CNT-HAP) for fluoride removal was investigated. The synthesized CNT-HAP composite was systematically characterized by X-ray diffraction(XRD), Fourier Transform infrared spectroscopy(FTIR), scanning electron microscope (SEM) and Brunauer–Emmett–Teller(BET). Batch adsorption experiments were conducted to investigate the defluorination capacity of CNT-HAP. The CNT-HAP composite has a maximum adsorption capacity of 11.05 mg·g^−1^ for fluoride, and the isothermal adsorption data were fitted by the Freundlich model to calculate the thermodynamic parameters. Thermodynamic analysis implies that the adsorption of fluoride on CNT-HAP is a spontaneous process. Furthermore, the adsorption of fluoride follows pseudo-second-order model. The effects of solution pH, co-existing anions and reaction temperature on defluorination efficiency were examined to optimize the operation conditions for fluoride adsorption. It is found that the optimized pH-value for fluoride removal by CNT-HAP composite is 6. In addition, among five common anions studied in this work, the presence of ${\text{HCO}}_{3}^{-}$ and ${\text{PO}}_{4}^{3-}$ could considerably affect the fluoride removal by CNT-HPA in aqueous media. Finally, the underlying mechanism for the fluoride removal by CNT-HAP is analyzed, and an anion exchange process is proposed.

## Introduction

Fluoride is an essential element for both human and animals. However, it may be useful or harmful to human bodies, depending on its concentration in drinking water and total ingested amount (Chen et al., 2012; Sharma et al., 2017). It is recommended by World Health Organization (WHO) that the most appropriate concentration of fluoride in drinking water is 0.5–1.5 mg·L^−1^, exceeding which people could have dental and / or skeletal fluorosis such as softening of bones, mottling of teeth and neurological damage (Liu et al., 2015; Zhang L. E. et al., 2017). Unfortunately, the Unfortunately, the concentration of fluoride has been found to be as high as 30 mg·L^−1^ in the drinking water of about 25 countries across the world, including India, Mexico and China (Amini et al., 2008). And the fluoride pollution in drinking water is even worse and worse. (Jagtap et al., 2012; Roy and Dass, 2013). Therefore, it is necessary to remove the excess fluoride from drinking water.

Currently, there are several methods available for defluorination, such as chemical precipitation (Xin et al., 2016; Huang et al., 2017), membrane filtration (Zhang J. et al., 2017), electrolysis (Schaefer et al., 2017), ion exchange (Popat et al., 1994; Jamhour, 2005), and adsorption (Rehman et al., 2015; Lin et al., 2016). Among the above-mentioned methods, adsorption is a most attractive option owning to its low cost, high efficiency, and good flexibility (Mohan et al., 2017). Hydroxyapatite [Ca_10_(PO_4_)_6_(OH)_2_, HAP] is a natural mineral with abundance in bone and skeletal tissues. It has been demonstrated to be a promising candidate adsorbent for fluoride removal because of its easy synthesis, high recyclability, and good biocompatibility (Beladi et al., 2017; Lei et al., 2017). Jiménez-Reyes. et al. reported that the defluorination capacity of HAP was 4.7 mg of fluoride/g of adsorbent in the pH range of 5.0–7.3 (Jiménez-Reyes and Solache-Ríos, 2010). Sundaram et al. prepared the HAP / chitosan nanocomposite for fluoride removal with a defluorination capacity of 1.56 mg·g^−1^ (Gao et al., 2009). There are two factors contributing to the removal of fluoride by HAP: ion exchange and electrostatic interaction (He et al., 2015). In this process, fluorapatite [Ca_5_(PO_4_)_3_F] or mixed fluorinated HAP [Ca_5_(PO_4_)_3_(OH-F)] which are thermodynamically more stable are formed.

However, the practical application of HAP in fluoride removal from drinking water is still limited by its low defluorination capacity. It has been well established that adsorbents with high surface areas and active sites density can be fabricated by tailoring the morphology and pore size (Chen et al., 2016). Moreover, adsorbents with hierarchically meso- and /or macroporous networks enable the fast diffusion of guest molecules in the channels, thus facilitating their access to active sites. Among various kinds of materials, carbon nanotubes (CNT) are particularly attractive because they not only have the characteristics mentioned above, but also can act as adsorbents for fluoride removal. For example, Li et al. (2003) reported use of aligned CNT to remove fluoride, and an adsorption capacity of 4.5 mg·g^−1^ was achieved in the aqueous solution with the fluoride concentration of 15 mg·L^−1^.

In a very recently study, Neelgund et al. (Neelgund and Oki, 2016) found that CNT could improve the photothermal efficiency (PTE) of HAP, and HAP could also overcome the poor dispersion of CNT. In this work, we proposed the combination of HAP with CNT for fluoride removal from aqueous media. To the best of our knowledge, there are few study on such a subject. The major objectives of our work are: (1) to characterize CNT-HAP composite by XRD, FTIR, SEM, and TGA; (2) to compare the defluorination performance between HAP and CNT-HAP composite;(3) to optimize the operation conditions for fluoride removal; (4) to disclose the underlying mechanism of fluoride removal.

## Experimental section

### Materials

All the chemicals were purchased from Tianjin Damao Chemicals Co. Ltd., (China), and used directly without further purification. The water used in this work was in ultrapure grade.

### Preparation of CNT-HAP composite

CNT-HAP composite was synthesized by co-precipitation. The pH of aqueous solutions of Ca(NO_3_)_2_ (0.25 mol·L^−1^) and (NH_4_)_2_HPO_4_ (0.3·mol L^−1^) (Ca/P = 1.67) were adjusted to 10.0 by NH_3_·H_2_O. The CNT (5 wt‰) was functionalized by HNO_3_, and then added into the aqueous solution of Ca(NO_3_)_2_. The solution was ultrasonicated until a homogeneous solution was formed (A). The aqueous solution of (NH_4_)_2_HPO_4_ was dripped slowly into the solution A under vigorous stirring for 1 h at 45°C to generate precipitates. Afterwards, the pH of solution was adjusted to 10.0 by NH_3_·H_2_O. The mixture was aged for 24 h at room temperature to form colloids. The colloids were centrifuged, washed with ultrapure water for several times, milled with ethanol, dried at 80°C, and finally calcined at 200°C for 2 h to give CNT-HAP composite.

### Materials characterizations

The phase composition and crystal structure of CNT-HAP composite were characterized by an X-ray powder diffractometer (XRD, D8 ADVANCE X), using Cu Kα (40 kV, 40 mA) radiation in the scanning range of 10–80°. The chemical structure was characterized by a Fourier transform infrared spectrometer (FTIR, Nicolet 5700,) in the wavenumber range of 400–4,000 cm^−1^. The elemental composition was examined by an X-ray photoelectron spectroscopy (XPS, Axis Ultra DLD), using Al Kα radiation. The morphology was examined by a scanning electron microscope (SEM, JSM 6701F) at the accelerating voltage of 5 kV. The specific surface area was calculated by Brunauer-Emmett-Teller (BET) equation from the N_2_ adsorption-desorption isotherms determined by a porosity analyser (JW-BK132F). The pH-values were determined by a pH electrode (pH SJ-4A). The concentrations of fluoride in aqueous solutions were determined by ion chromatography (IC, ICS-1100).

### Adsorption experiments

The adsorption experiments were performed by the batch method to investigate the effects of different parameters (e.g., temperature, reaction time, pH and co-existing ions) on fluoride removal by CNT-HAP. In a typical run, 0.01 g of CNT-HAP was mixed with 20 mL of aqueous solution containing fluoride, and the mixture was stirred at the rate of 180 rpm and temperature of 25°C for 24 h to achieve the adsorption equilibrium.

## Results and discussion

### X-ray diffraction

**Figure 1:**
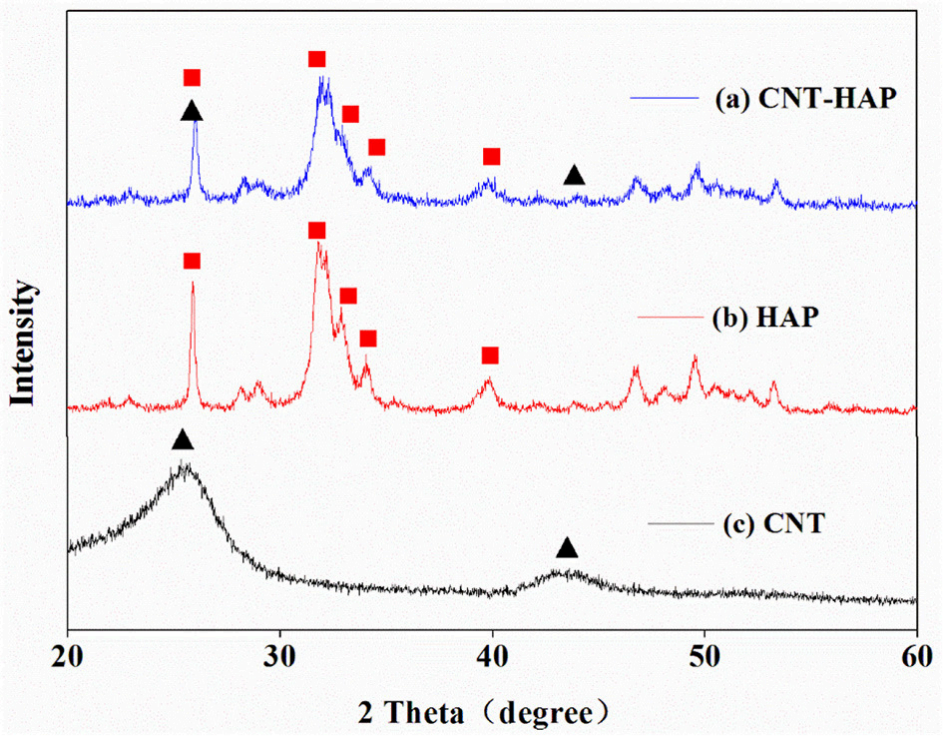
XRD patterns of CNT-HAP **(a)**, HAP **(b)**, and CNT **(c)**.

The XRD patterns of HAP, CNT and CNT-HAP composite are presented in Figure 1. The diffraction peaks of HAP could be indexed to hexagon-phased HAP with considerable intensities at 26, 33, 34, 35, and 40° (JCPDS file 09-0432). In comparison with the diffraction patterns of HAP, those of CNT-HAP are very similar, indicating the successful incorporation of HAP into the matrices of CNT. However, it is worth noting that the diffraction patterns of CNT could hardly be observed in those of CNT-HAP composite, which should be attributed to the overlap of the major diffraction peaks of CNT and HAP at 26°. On the other hand, if CNT was directly wrapped by as-synthesized HAP, the characteristic peaks of CNT can also not be observed in the diffraction patterns of CNT-HAP composite. This could also elucidate successful synthesis of CNT-HAP. In addition, the sharp and symmetric diffraction peaks confirm good crystallinity of synthesized CNT-HAP composite.

### Infrared spectroscopy

**Figure 2:**
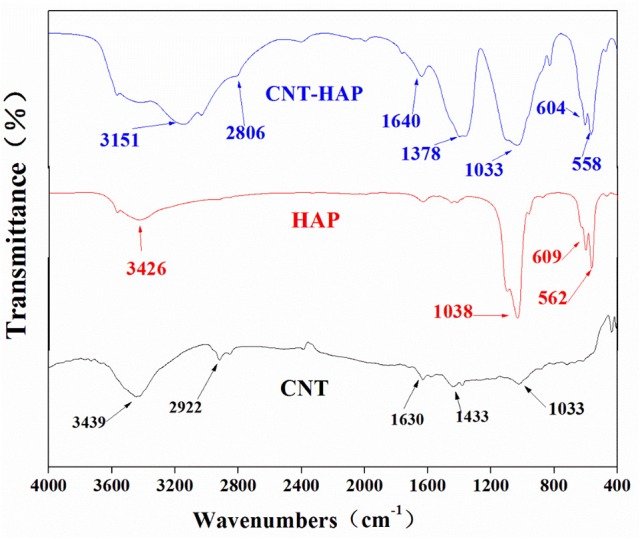
FT-IR spectra of CNT, HAP, and CNT-HAP.

The FT-IR spectra of HAP, CNT and CNT-HAP are shown in Figure 2. The broad peaks at 3,150–3,550 cm^−1^ are assigned to the O-H vibration (Xu et al., 2013). The bands at 1,030–1,040, 600–610, and 550–570 cm^−1^ are associated with the stretching vibration of phosphate, confirming the presence of HAP in CNT-HAP composite. Besides those peaks associated with HAP, the C-H stretching vibration appears at 2,800–3,000 cm^−1^ while the C-H bending vibration is observed at 1,370–1,400 cm^−1^ (Zhang et al., 2012). The peaks at 1,630–1,640 cm^−1^ are attributed to the C = O stretching vibration. All these characteristic peaks can also be found in the acidified CNT, confirming the pliable phase composition of CNT-HAP composite, which is in accordance with the results concluded from XRD patterns.

### Scanning electron microscopy

**Figure 3:**
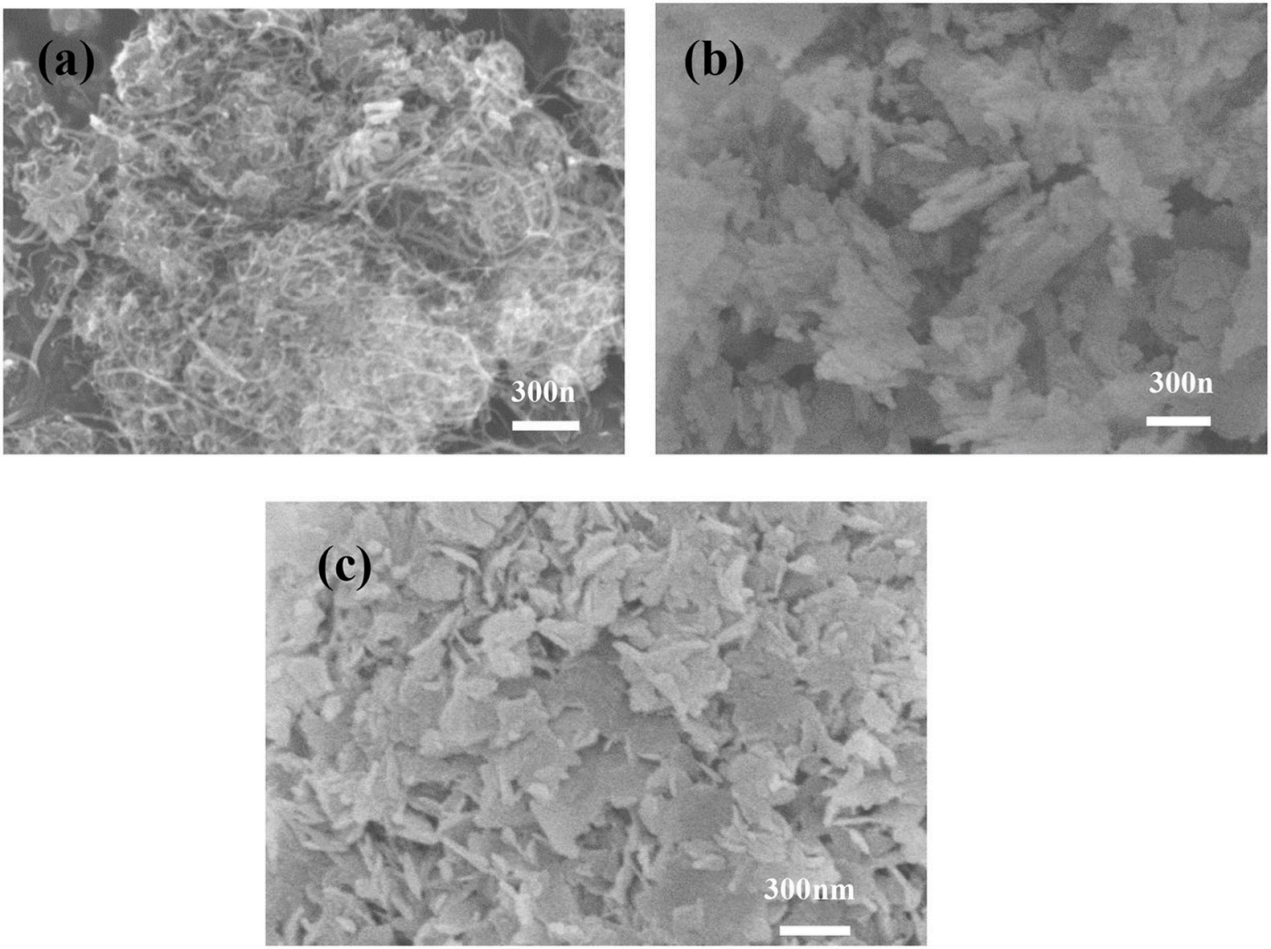
SEM images of CNT **(a)**, HAP **(b)**, and CNT-HAP **(c)**.

The SEM images of CNT, HAP and CNT-HAP are presented in Figure 3. HAP displays faint lamellar morphology and compact internal structure, while CNT-HAP displays legible lamellar morphology because the introduction of CNT is beneficial for the nucleation and crystallization of HAP. The vertical growth of HAP along CNT implies that CNT-HAP has higher specific surface area than HAP. In Figure 3c, the characteristic tubular structure cannot be observed in CNT-HAP, suggesting that CNT is wrapped by HAP on the surface, which is consistent with the results concluded from XRD patterns. The SEM images proves the successful synthesis of CNT-HAP and the assembly of HAP on the surface of CNT.

### Adsorption isotherms

**Figure 4:**
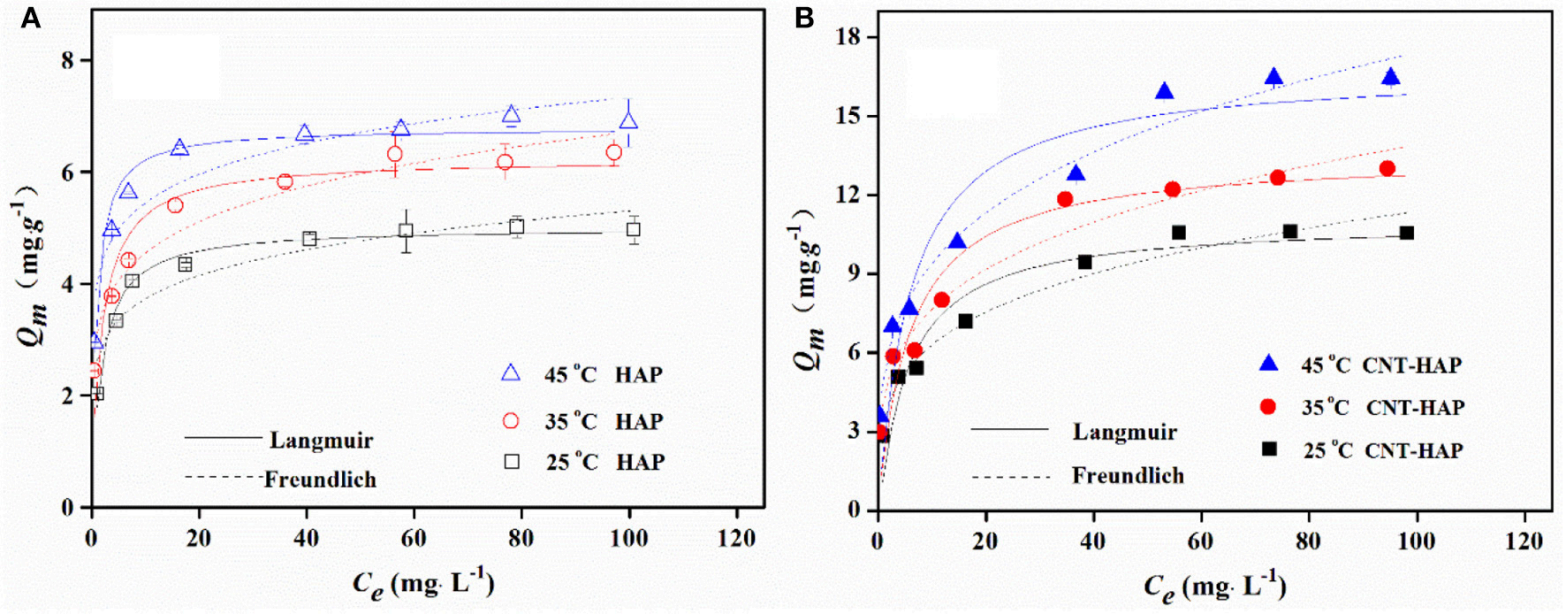
Adsorption isotherms of fluoride on HAP **(A)** and CNT-HAP **(B)** at three different temperatures (adsorbents dosage: 0.5 g·L^−1^, pH: 7.0).

The adsorption isotherms of fluoride on HAP and CNT-HAP at 25, 35, and 45°C were determined, as shown in Figure 4. The adsorbed amounts of fluoride by HAP and CNT-HAP both increase rapidly at low fluoride concentrations, and the increasing trend gradually fades out at high fluoride concentrations. With the increase of temperature, the adsorbed amounts of fluoride increase continuously. The maximum adsorption capacity (*q*_*m*_) of fluoride on CNT-HAP at 25°C is 11.05 mg·g^−1^, being higher than pristine HAP and other HAP-based materials reported in the literature (Table 1; Gao et al., 2009; Sairam Sundaram et al., 2009; Jiménez-Reyes and Solache-Ríos, 2010; Liu et al., 2010; Kanno et al., 2014; Prabhu and Meenakshi, 2014; He et al., 2017; Nigri et al., 2017; Zúñiga-Muro et al., 2017).

**Table 1:** Comparison of fluoride adsorption capacities of pristine HAP and various HAP-based materials.

**Adsorbents**	**Adsorption capacities (mg·g^−1^)**	***C*_initial_ (mg·L^−1^)**	**pH**	**References**
HAP powder	4.7	20	7.0	Jiménez-Reyes and Solache-Ríos, 2010
Nano-HAP/Chitin	3.0	10	7.0	Sairam Sundaram et al., 2009
Al-HAP adsorption membrane	7.15	10	7.0	He et al., 2017
HAP-coated-limestone	9.3	50	7.0	Kanno et al., 2014
Synthetic nano-HAP	4.8	80	5.0–6.0	Gao et al., 2009
DTAB-HAP powder	3.436	10	7.0	Prabhu and Meenakshi, 2014
Nano-HAP/Chitosan	1.56	10	7.0	Sairam Sundarama, 2008
Synthetic siderite	1.775	3–20	4.0–9.0	Liu et al., 2010
bone char	4.81	10	7.2–7.7	Nigri et al., 2017
cerium-containing bone char	13.6	10	7.0	Zúñiga-Muro et al., 2017

To better understand the adsorption behavior of fluoride, the Langmuir and Freundlich models were used to correlate the adsorption data. The Langmuir model (Equation 1) applicable for monolayer adsorption process (Li et al., 2012; Reynel-Avila et al., 2016).

(1) $$\begin{array}{r} q_{e}=\frac{q_{m}k_{L}c_{e}}{1+k_{L}c_{e}} \end{array}$$

Where *q*_*e*_ (mg·g^−1^) is the adsorbed amount of fluoride at equilibrium; *Ce* (mg·L^−1^) (mg·L^−1^) is the concentration of fluoride in supernatant solution at equilibrium; *q*_*m*_ (mg·g^−1^) represents the maximum adsorption capacity; and *k*_*L*_ (L·mg^−1^) represents the adsorption equilibrium constant. The Freundlich model (Equation 2) is applicable for the description of adsorption data on heterogeneous surfaces at low to intermediate concentrations (Mahmoud et al., 2016).

(2) $$\begin{array}{r} q_{e}=k_{F}{C_{e}}^{\frac{1}{n}} \end{array}$$

Where *k*_*F*_ (mg^1−n^·L^n^·g^−1^) i the adsorption capacity when the equilibrium concentration of ion equals to 1; and *n* is the dependent degree of adsorption capacity on equilibrium concentration, with the value of 1~10. The fitted parameters for two adsorption models as well as the correlation coefficients (*R*^2^) are shown in Table 2. According to the values of *R*^2^ and fitted curves shown in Figure 4, it is obvious that the Langmuir model is better for describing the adsorption behavior of fluoride on HAP, implying that the adsorption energy is constant and the adsorption capacity is limited by the amounts of active sites. In contrast, the Freundlich model is better for describing the adsorption behavior of fluoride on CNT-HAP, implying that the adsorption of fluoride is subjected to multi-layer and physical-chemical adsorption.

**Table 2:** Fitted Langmuir and Freundlich model parameters for fluoride adsorption on HAP and CNT-HAP composite at different temperatures.

**Samples**	**Temperatures (°C)**	**Langmuir model**			**Freundlich model**		
		***q_*m*_* / (mg·g^−1^)**	***K_*L*_* / (L·mg^−1^)**	***R^2^***	***k_*F*_***	***n***	***R^2^***
HAP	25	5.01	0.55	0.9772	2.63	6.56	0.8932
HAP	35	6.25	0.50	0.9493	3.07	5.88	0.9136
HAP	45	6.81	1.08	0.9479	4.07	7.77	0.8933
CNT-HAP	25	11.05	0.18	0.9142	3.57	3.96	0.9735
CNT-HAP	35	13.57	0.17	0.9075	4.26	3.88	0.9695
CNT-HAP	45	16.78	0.17	0.8835	5.08	3.74	0.9831

### Adsorption thermodynamic analysis

The thermodynamic parameters such as Gibbs free energy change (Δ*G*°), adsorption enthalpy (Δ*H*°) and adsorption entropy (Δ*S*°) could be calculated from the adsorption data according to eqs. 3~6 (Luo et al., 2015; Wang et al., 2015; Singh and Anil Kumar, 2016).

(3) $$\begin{array}{r} \Delta G^{{}^{\circ}}=-RTlnK_{d} \end{array}$$

(4) $$\begin{array}{r} \Delta S^{{}^{\circ}}=-{\left(\frac{\partial \Delta G^{{}^{\circ}}}{\partial T}\right)}_{P} \end{array}$$

(5) $$\begin{array}{r} \Delta H^{{}^{\circ}}=\Delta G^{{}^{\circ}}+T\Delta S^{{}^{\circ}} \end{array}$$

(6) $$\begin{array}{r} \text{InK}=\Delta {\text{S}}^{{}^{\circ}}/\text{R}-\Delta {\text{H}}^{{}^{\circ}}/\text{RT} \end{array}$$

Where *R* is the gas constant (8.314 J·mol^−1^·K^−1^); T is the absolute temperature (K); and *K*_*d*_ is the adsorption equilibrium constant. As shown in Table 3, the negative values of ΔG° indicate that the adsorption process of fluoride is spontaneous. The much lower values of ΔG°For fluoride adsorption on CNT-HAP than on HAP at the same temperature suggests the easier adsorption of fluoride on CNT-HAP than on HAP. The positive value of ΔS° elucidate that the organization of fluoride ions on the surface of adsorbents is more random than in the aqueous solutions. The positive values of ΔH° suggest that the adsorption of fluoride on HAP and CNT-HAP is endothermic.

**Table 3:** The thermodynamic parameters of fluoride adsorption on HAP and CNT-HAP composite.

**Adsorbents**	***T*(°C)**	**Δ*G^0^* (kJ·mol^−1^)**	**Δ*H^0^* (kJ·mol^−1^)**	**Δ*S^0^* (J·mol^−1^ ·K^−1^)**	**ln*K_*d*_***
CNT-HAP	25	−16.53			6.67
CNT-HAP	35	−17.78	20.68	124.38	6.94
CNT-HAP	45	−19.03			7.19
HAP	25	−15.54	19.80	118.72	6.27
HAP	35	−16.81			6.56
HAP	45	−17.90			6.77

### Adsorption kinetics

The adsorbed amounts of fluoride were plotted vs. adsorption time to examine the adsorption kinetics, as shown in Figure 2. The amounts of fluoride adsorbed by HAP and CNT-HAP rapidly increase in the first 2 h. Then the adsorption rate slows down and reaches equilibrium at 240 and 300 min, respectively. The adsorption kinetic data were fitted by pseudo-first-order model (Equation 7), and pseudo-second-order model (Equation 8), respectively (Naowanat et al., 2016; Subbaiah and Kim, 2016; Sun et al., 2017).

(7) $$\begin{array}{r} q_{t}=q_{e}\left(1-e^{{-k}_{1}t}\right) \end{array}$$

(8) $$\begin{array}{r} q_{r}=\frac{q_{e}^{2}k_{2}t}{1+q_{e}k_{2}t} \end{array}$$

**Figure 5:**
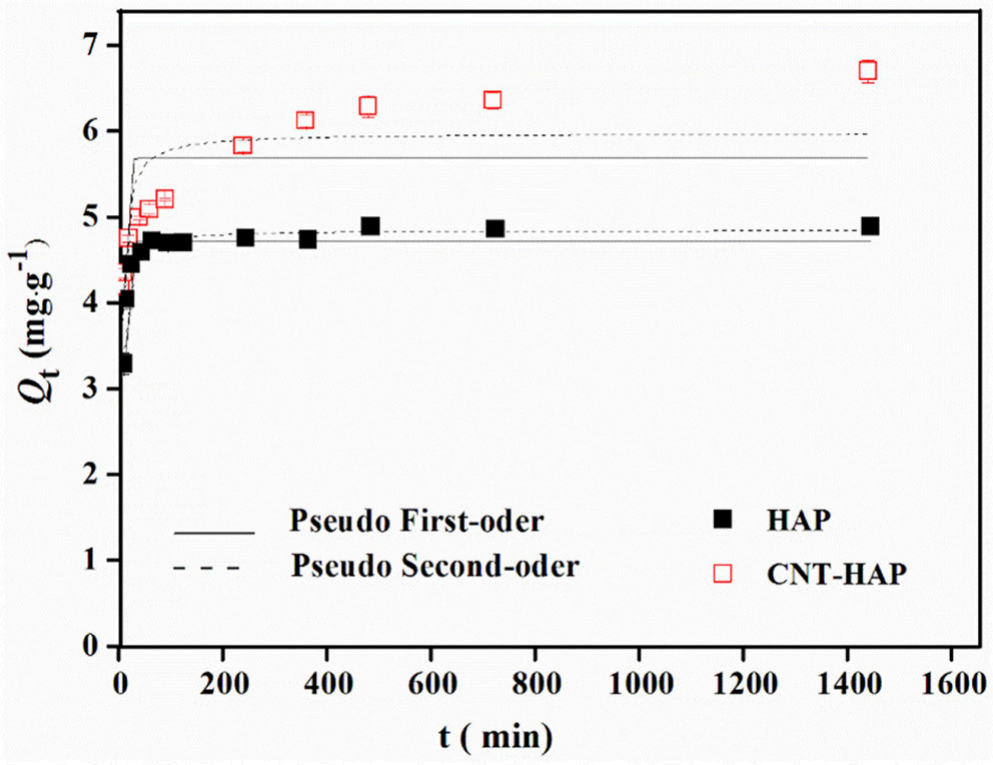
Nonlinear fitting for kinetic data of fluoride adsorption on HAP and CNT-HAP.

Where *k*_1_ (min^−1^); *k*_2_ (g·mg^−1^·h^−1^) are the pseudo-first-order and the pseudo-second-order rate constant, respectively; *q*_*t*_ (mg·g^−1^) is the adsorbed amount of fluoride on adsorbents at time t (min); *q*_*e*_ (mg·g^−1^) is the equilibrium adsorption capacity. The nonlinear fitting for *q*_*t*_ vs. *t* is presented in Figure 5. All the fitted kinetic kinetic parameters as well as the correlation coefficients (*R*^2^) are listed in Table 4. Obviously, the kinetic data of fluoride adsorption on HAP and CNT-HAP are well fitted to the pseudo-second-order model, indicating that the fluoride removal processes are subjected to chemical reaction (Tadjarodi et al., 2016), which determines the overall adsorption rate.

**Table 4:** Fitted kinetic parameters for fluoride adsorption on HAP and CNT-HAP composite.

**Samples**	**Pseudo first-order**			**Pseudo second-order**		
	***k*_1_ / (min^−1^)**	***q*_*e*_ / (mg·g^−1^)**	***R*^2^**	***k*_2_ / (g·mg^−1^·min^−1^)**	***q_*e*_* / (mg·g^−1^)**	***R^2^***
HAP	0.22	4.73	0.9204	0.09	4.85	0.9827
CNT-HAP	0.20	5.70	0.3956	0.05	5.97	0.8277

### Effects of solution pH and co-existing anions

**Figure 6:**
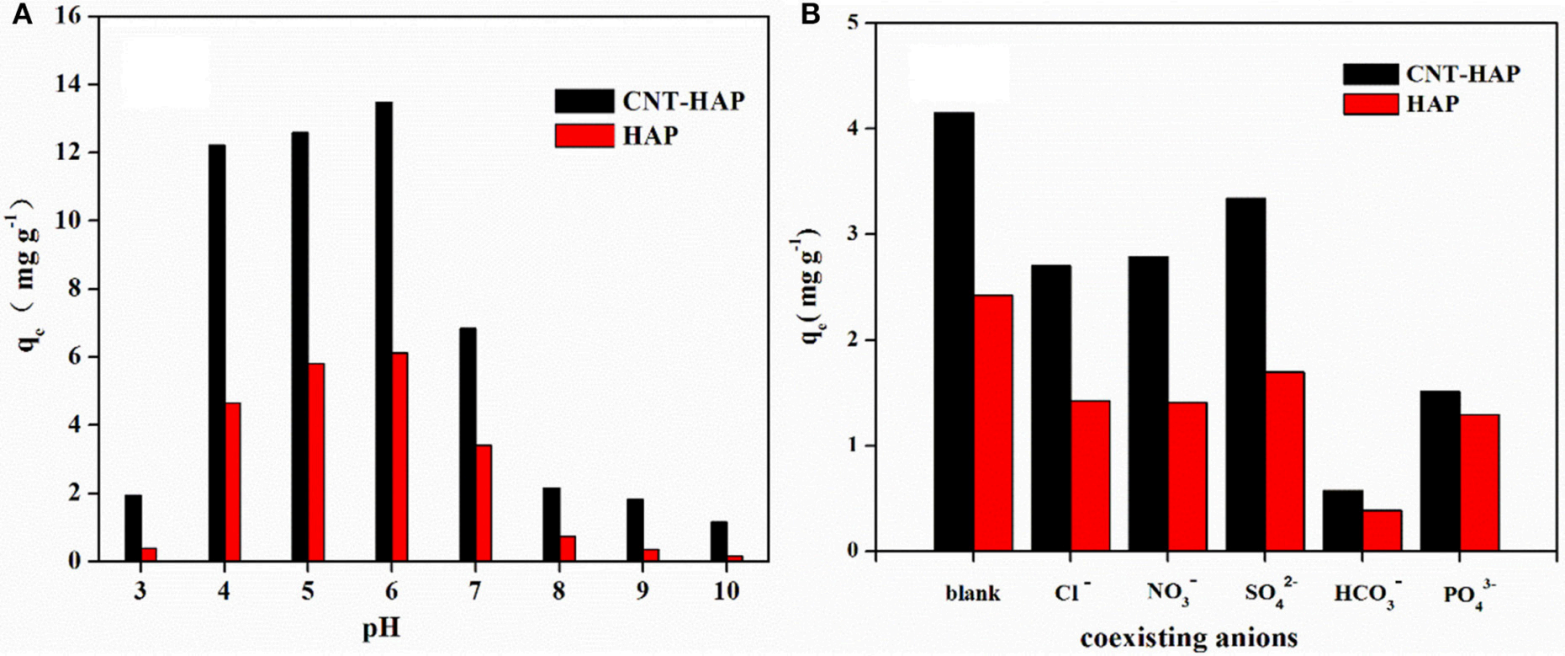
Effects of pH (3.0–11.0) **(A)** and coexisting anions **(B)** on the removal of fluoride by HAP and CNT-HAP (adsorbents dosage: 0.5 g·L^−1^, *C*_0_ = 10 mg·L^−1^, reaction time = 24 h, temperature = 25°C).

The adsorption of fluoride on HAP and CNT-HAP from aqueous solutions with different pH values ranging from 3.0 to 10.0 was investigated (Figure 6A). Obviously, the CNT-HAP composite exhibits considerably higher adsorption capacities for fluoride than HAP, thereby displaying great potential application in defluorination from aqueous media. As for the effect of solution pH, the fluoride adsorption capacities first increase in the pH range of 3.0–6.0, but then decrease in the pH range of 6.0–10.0. Therefore, the optimal pH-value for fluoride removal is 6.0. Under such a weakly acidic condition, the surface of CNT-HAP and HAP would be protonated, thus increase the density of active sites on the surface (Jiménez-Reyes and Solache-Ríos, 2010; Nie et al., 2012). In contrast, the surface of CNT-HAP and HAP would be saturated with negative charges under alkaline condition, which restrains the diffusion of fluoride ions on the surface, thus resulting in lower adsorption capacities. Our results are consistent with some other works, which reported the high fluoride removal efficiency in acidic media owing to the attraction of fluoride anion to the positively charged adsorbents surface, and the low fluoride removal efficiency in alkaline media due to the repulsion of fluoride anion from the negatively charge adsorbents surface.

In addition, the removal of fluoride in the presence of five co-existing anions (Cl^−^, ${\text{NO}}_{3}^{-}$, ${\text{HCO}}_{3}^{-}$, ${\text{SO}}_{4}^{2-}$, and ${\text{PO}}_{4}^{3-}$) were investigated, and results are shown in Figure 6B. As can be seen, the introduction of Cl^−^, ${\text{NO}}_{3}^{-}$, and ${\text{SO}}_{4}^{2-}$ have slightly negative effect on the fluoride removal efficiency, while the introduction of ${\text{HCO}}_{3}^{-}$ and ${\text{PO}}_{4}^{3-}$ has significantly negative impact. This phenomenon can be explained by the charge/radius (Z/r, Å) of these anions. In our case, the order of Z/r values of some common anions is ${\text{PO}}_{4}^{3-}$ (3/2.38) > ${\text{SO}}_{4}^{2-}$ (2/2.30) > F^−^ (1/1.33) > OH^−^ (1/1.37) > ${\text{HCO}}_{3}^{-}$(1/1.56) > NO_3_ (1/1.79) > Cl (1/1.81) (Yang et al., 2014). Clearly, the Z/r value of ${\text{PO}}_{4}^{3-}$ is the largest among these anions, suggesting that ${\text{PO}}_{4}^{3-}$ could easily form bond with Ca^2+^ in competition with F^−^, and reduce the fluoride adsorption capacities (Nie et al., 2012). The Z/r value of ${\text{HCO}}_{3}^{-}$ (1/1.56) is similar to that of OH^−^ (1/1.37), as a result, it could replace the OH^−^ in HAP, which subsequently influence the fluoride adsorption capacities. ${\text{SO}}_{4}^{2-}$, though with high Z/r value as well, has only slight effect on the fluoride adsorption capacities because of its large ionic radii (2.30 Å). Cl^−^ and ${\text{NO}}_{3}^{-}$ also have only slight influence on the fluoride adsorption capacities because of they have lower ability for binding with the active sites on adsorbents than F^−^ (Mohanty et al., 2005).

### Brunauer-Emmett-Teller analysis

The BET surface area of CNT-HAP composite was determined to be 70.94 m^2^·g^−1^, being much larger than CNT and HAP (32.60 and 20.78 m^2^·g^−1^), which is consistent with the results concluded from SEM images. The enhancement of specific surface area in CNT-HAP composite can be explained by the vertical growth of HAP on the surface of CNT, although they are not assembled compactly. Consequently, the CNT-HAP composite exhibits improved internal density.

**Figure 7:**
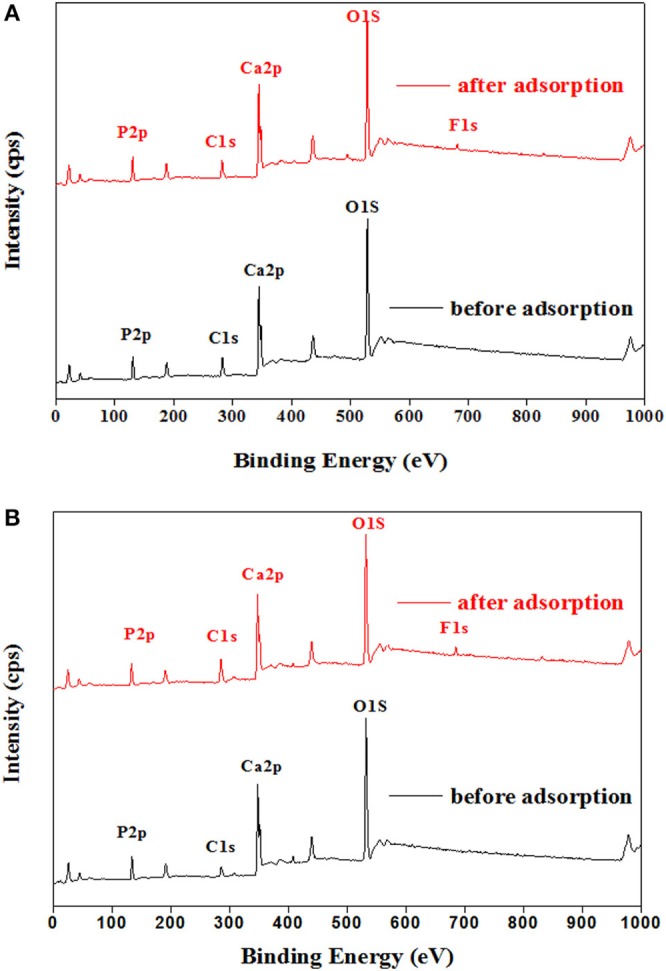
XPS spectra of the HAP **(A)** and CNT-HAP **(B)** before and after fluoride adsorption.

Generally, the fluoride removal by HAP is subjected to ion exchange, during which the original hydroxyl anions attached to HAP are replaced by fluoride anions (Chen et al., 2016). To examine the reaction mechanism for fluoride adsorption on CNT-HAP, the XPS spectra of HAP and CNT-HAP before and after fluoride adsorption were collected and shown in Figure 7. As shown in Figure 7A, the F 1s signal locating at 680 eV can be observed in the XPS spectra of HAP after fluoride adsorption, indicating that fluoride is binding to the surface of HAP. In the XPS spectra shown in Figure 7B, the F 1s signal locating at 680 eV also appears after fluoride adsorption on CNT-HAP, which is similar to the case of fluoride adsorption on HAP. Therefore, we confirm that the fluoride removal by CNT-HAP is also subjected to anion exchange mechanism.

**Figure 8:**
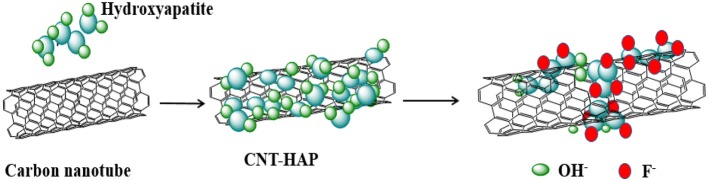
Illustration of the underlying mechanism for fluoride removal by CNT-HAP.

Based on the results obtained, the underlying mechanism for fluoride removal by CNT-HAP is proposed and depicted in Figure 8. First, the HAP is doped with CNT via co-precipitation method to produce CNT-HAP composite. The HAP is mainly loaded on the surface of CNT, and the CNT-HAP composite is of large surface area. Then the hydroxyl anions in HAP adsorb fluoride via ion exchange. The hydroxyl ion in solution will increases the pH of the bath solution during the experiments, thus the reaction was suitable to occur in a weakly acidic condition, which was consistent with the result showed above. In summary, it is suggested that the significantly enhance fluoride removal efficiency of CNT-HAP is attributed to the cooperative effect of surface hydroxyl anions in HAP and large surface area of CNT.

## Conclusion

The hydroxyapatite decorated with carbon nanotube was prepared and used as an effective adsorbent for fluoride removal. The adsorption of fluoride on CNT-HAP could be well described by the Freundlich model. The nature of the adsorption process is spontaneous and endothermic. The kinetics of adsorption follows pseudo-second-order. The defluorination capacity can be significantly affected by the solution pHand co-existing anions. Combining the results from XRD, FTIR, SEM, BET, and XPS analysis, it is demonstrated that the adsorption mechanism follows an anion exchange process. The efficiency of fluoride removal by CNT-HAP is greatly enhanced in relative to the pristine HAP, because the introduction of CNT can enlarge the specific surface area of HAP, thereby affording more surface hydroxyl anions to be replaced by fluoride.

## Author contributions

QT: Performed the experiments and the data analyses and wrote the manuscript; TD: Helped performed the experiments and analyze the data; PL: Revised the manuscript; PZ: Coordinated and supervised the research activities that are being described in the manuscript, and DW: Contributed to the conception of this study.

### Conflict of interest statement

The authors declare that the research was conducted in the absence of any commercial or financial relationships that could be construed as a potential conflict of interest.
